# Supplementary material for: Association of missense variant DCLRE1B rs3761936 with breast and cervical cancer risk—A case-control study
Source: PLoS One. 2025 Sep 8;20(9):e0331492. doi: 10.1371/journal.pone.0331492 (PMC12416678; doi:10.1371/journal.pone.0331492)
Supplement: S1 Table — (DOCX) [file pone.0331492.s001.docx]

**Table S1: Primer sequences used for amplification**

| *Gene* | *SNP* | *Primer sequence (5’-3’)* | *PCR Product* | *Tm (℃)* |
| --- | --- | --- | --- | --- |
| *DCLRE1B* | rs3761936 | FI: AAGCCCTGGAGGTTGGTGAGAGCAAT  RI: TCTTGTCCAATTTCATCTAGGGGTAATCCG  FO: ATTGCTTGGGAATCCCCATTACAGCTTC  RO: GAGAAGTCACTGGGATTCCTTGAGAGGC | 180 bp  220 bp  344 bp | 64  59.6  61  61.9 |
