# Supplementary material for: Association of missense variant DCLRE1B rs3761936 with breast and cervical cancer risk—A case-control study
Source: PLoS One. 2025 Sep 8;20(9):e0331492. doi: 10.1371/journal.pone.0331492 (PMC12416678; doi:10.1371/journal.pone.0331492)
Supplement: S2 Table — (DOCX) [file pone.0331492.s002.docx]

**Table S2: Distribution of genotype and allelic frequencies of *DCLRE1B* rs3761936 polymorphism between cancer patients and control population**

| ***DCLRE1B***  ***rs3761936*** | ***BC Cases***  ***n=135 (%)*** | ***HWE*** | | ***CC Cases***  ***n=110 (%)*** | ***HWE*** | | ***Controls***  ***n=108 (%)*** | ***HWE*** | |
| --- | --- | --- | --- | --- | --- | --- | --- | --- | --- |
|  |  | ***χ^2^*** | ***p-value*** |  | ***χ^2^*** | ***p-value*** |  | ***χ^2^*** | ***p-value*** |
| **TT** | 57 (42.22) | 0.02 | 0.891 | 53 (48.18) | 0.27 | 0.601 | 70 (64.81) | 0.19 | 0.664 |
| **TC** | 62 (45.93) |  |  | 45 (40.91) |  |  | 33 (30.56) |  |  |
| **CC** | 16 (11.85) |  |  | 12 (10.91) |  |  | 5 (4.63) |  |  |
| **T** | 176 (65.18) |  |  | 151 (68.64) |  |  | 173 (80.09) |  |  |
| **C** | 94 (34.81) |  |  | 69 (31.36) |  |  | 43 (19.91) |  |  |
