# Supplementary material for: Association of missense variant DCLRE1B rs3761936 with breast and cervical cancer risk—A case-control study
Source: PLoS One. 2025 Sep 8;20(9):e0331492. doi: 10.1371/journal.pone.0331492 (PMC12416678; doi:10.1371/journal.pone.0331492)
Supplement: Attachment — Submitted filename: PONE-D-23-33713.pdf [file pone.0331492.s004.pdf]

PLOS ONE

The association of missense variant DCLRE1B rs3761936 with breast and cervical cancer risk - A case-control study

--Manuscript Draft--

**Manuscript Number:**	PONE-D-23-33713
**Article Type:**	Research Article
**Full Title:**	The association of missense variant DCLRE1B rs3761936 with breast and cervical cancer risk - A case-control study
**Short Title:**	DCLRE1B rs3761936 polymorphism and breast and cervical cancer risk
**Corresponding Author:**	Mohammad Safiqul Islam, Ph.D<br>Noakhali Science and Technology University Faculty of Pharmacy<br>Noakhali, BANGLADESH
**Keywords:**	DCLRE1B; breast cancer; cervical cancer; polymorphism; rs3761936; T-ARMS PCR
**Abstract:**	Background: Overexpression of rs3761936 of DCLRE1B gene has been observed in both breast cancer and cervical cancer patients. To justify the association of this polymorphism with these cancers, we performed this case-control study.<br>Method: A total of 245 cancer patients and 108 healthy controls participated in the research. An efficient T-ARMS PCR method was used for genotyping.<br>Results: The cancer patients showed higher mutant allele frequency compared to the controls. Mutant allele carrier breast cancer patients showed significantly increased risk in four genetic models, including additive model 1 (TC vs. TT: OR=2.31, 95% CI=1.33-3.99, p-value=0.0028), additive model 2 (CC vs. TT: OR=3.93, 95% CI=1.36-11.38, p-value=0.0116), dominant model (TC+CC vs. TT: OR=2.52, 95% CI=1.50-4.25, p-value=0.0005), and over-dominant model (TC vs. TT+CC: OR=1.93, 95% CI=1.13-3.28, p-value=0.0152). The allele frequency analysis showed that mutant allele C carriers among breast cancer patients had a significantly higher risk than the wild type T allele carriers (C vs. T: OR=2.15, 95% CI=1.41-3.26, p-value=0.0003). Likewise, the cervical cancer patients showed significant risk in three genetic models, including additive model 1 (TC vs. TT: OR=1.80, 95% CI=1.01-3.20, p-value=0.0444), additive model 2 (CC vs. TT: OR=3.17, 95% CI=1.05-9.55, p-value=0.0403), and dominant model (TC+CC vs. TT: OR=1.98, 95% CI=1.15-3.41, p-value=0.0138). The mutant allele C carriers had a significantly higher risk than the wild-type T allele carriers (C vs. T: OR=1.84, 95% CI=1.19-2.85, p-value=0.0065).<br>Conclusion: DCLRE1B rs3761936 is strongly associated with breast cancer and cervical cancer risk in Bangladeshi women.
**Order of Authors:**	Sarah Jafrin
	Md. Abdul Aziz, M.Pharm
	Md Abdul Barek, M.Pharm
	Md.Sharif Reza, M.Pharm
	Nura Ershad Naznin
	Mohammad Safiqul Islam, Ph.D
**Additional Information:**	
**Question**	**Response**
**Financial Disclosure**<br><br>Enter a financial disclosure statement that describes the sources of funding for the work included in this submission. Review the submission guidelines for detailed requirements. View published research	Yes

Powered by Editorial Manager® and ProduXion Manager® from Aries Systems Corporation

articles from *PLOS ONE* for specific examples.<br><br>This statement is required for submission and **will appear in the published article** if the submission is accepted. Please make sure it is accurate.<br><br>**Funded studies**<br>Enter a statement with the following details:<br>• Initials of the authors who received each award<br>• Grant numbers awarded to each author<br>• The full name of each funder<br>• URL of each funder website<br>• Did the sponsors or funders play any role in the study design, data collection and analysis, decision to publish, or preparation of the manuscript?<br><br>Did you receive funding for this work?	
Please add funding details.<br>as follow-up to "**Financial Disclosure**<br><br>Enter a financial disclosure statement that describes the sources of funding for the work included in this submission. Review the submission guidelines for detailed requirements. View published research articles from *PLOS ONE* for specific examples.<br><br>This statement is required for submission and **will appear in the published article** if the submission is accepted. Please make sure it is accurate.	The Research Cell, Noakhali Science and Technology University, Noakhali-3814, Bangladesh, funded this study partially (NSTU/RC/20/C-86), and no other public or private logistic funding was provided

**Funded studies**<br>Enter a statement with the following details:<br>• Initials of the authors who received each award<br>• Grant numbers awarded to each author<br>• The full name of each funder<br>• URL of each funder website<br>• Did the sponsors or funders play any role in the study design, data collection and analysis, decision to publish, or preparation of the manuscript?<br><br>Did you receive funding for this work?"	
Please select the country of your main research funder (please select carefully as in some cases this is used in fee calculation).<br>as follow-up to "**Financial Disclosure**<br><br>Enter a financial disclosure statement that describes the sources of funding for the work included in this submission. Review the submission guidelines for detailed requirements. View published research articles from *PLOS ONE* for specific examples.<br><br>This statement is required for submission and **will appear in the published article** if the submission is accepted. Please make sure it is accurate.<br><br>**Funded studies**<br>Enter a statement with the following details:<br>• Initials of the authors who received each award<br>• Grant numbers awarded to each author<br>• The full name of each funder<br>• URL of each funder website<br>• Did the sponsors or funders play any role in the study design, data collection and analysis, decision to publish, or preparation of the manuscript?<br><br>Did you receive funding for this work?"	BANGLADESH - BD
**Competing Interests**	The authors have declared that no competing interests exist.

Powered by Editorial Manager® and ProduXion Manager® from Aries Systems Corporation

Use the instructions below to enter a competing interest statement for this submission. On behalf of all authors, disclose any competing interests that could be perceived to bias this work—acknowledging all financial support and any other relevant financial or non-financial competing interests.<br><br>This statement is required for submission and **will appear in the published article** if the submission is accepted. Please make sure it is accurate and that any funding sources listed in your Funding Information later in the submission form are also declared in your Financial Disclosure statement.<br><br>View published research articles from *PLOS ONE* for specific examples.<br><br>**NO authors have competing interests**<br><br>Enter: *The authors have declared that no competing interests exist.*<br><br>**Authors with competing interests**<br><br>Enter competing interest details beginning with this statement:<br><br>*I have read the journal's policy and the authors of this manuscript have the following competing interests: [insert competing interests here]*<br><br>* typeset	
**Ethics Statement**<br><br>Enter an ethics statement for this submission. This statement is required if the study involved:<br><br>• Human participants<br>• Human specimens or tissue<br>• Vertebrate animals or cephalopods<br>• Vertebrate embryos or tissues	The 1964 Helsinki Declaration, and its later revisions, or equivalent ethical standards, were followed in all procedures conducted in research involving human subjects. The Ethical Review Board of the National Institute of Cancer Research and Hospital approved the study protocol (for breast cancer: NICRH/Ethics/2019/446 and for cervical cancer: NICRH/Ethics/2019/447).

• Field research<br><br>Write "N/A" if the submission does not require an ethics statement.<br><br>General guidance is provided below. Consult the submission guidelines for detailed instructions. **Make sure that all information entered here is included in the Methods section of the manuscript.**	

Powered by Editorial Manager® and ProduXion Manager® from Aries Systems Corporation

**Format for specific study types**<br><br>**Human Subject Research (involving human participants and/or tissue)**<br>• Give the name of the institutional review board or ethics committee that approved the study<br>• Include the approval number and/or a statement indicating approval of this research<br>• Indicate the form of consent obtained (written/oral) or the reason that consent was not obtained (e.g. the data were analyzed anonymously)<br><br>**Animal Research (involving vertebrate animals, embryos or tissues)**<br>• Provide the name of the Institutional Animal Care and Use Committee (IACUC) or other relevant ethics board that reviewed the study protocol, and indicate whether they approved this research or granted a formal waiver of ethical approval<br>• Include an approval number if one was obtained<br>• If the study involved *non-human primates*, add *additional details* about animal welfare and steps taken to ameliorate suffering<br>• If anesthesia, euthanasia, or any kind of animal sacrifice is part of the study, include briefly which substances and/or methods were applied<br><br>**Field Research**<br><br>Include the following details if this study involves the collection of plant, animal, or other materials from a natural setting:<br>• Field permit number<br>• Name of the institution or relevant body that granted permission	
**Data Availability**<br><br>Authors are required to make all data underlying the findings described fully available, without restriction, and from the time of publication. PLOS allows rare exceptions to address legal and ethical concerns. See the PLOS Data Policy and FAQ for detailed information.	No - some restrictions will apply

A Data Availability Statement describing where the data can be found is required at submission. Your answers to this question constitute the Data Availability Statement and **will be published in the article**, if accepted.<br><br>**Important:** Stating 'data available on request from the author' is not sufficient. If your data are only available upon request, select 'No' for the first question and explain your exceptional situation in the text box.<br><br>Do the authors confirm that all data underlying the findings described in their manuscript are fully available without restriction?	
**Describe where the data may be found in full sentences. If you are copying our sample text, replace any instances of XXX with the appropriate details.**<br><br>• If the data are **held or will be held in a public repository**, include URLs, accession numbers or DOIs. If this information will only be available after acceptance, indicate this by ticking the box below. For example: *All XXX files are available from the XXX database (accession number(s) XXX, XXX.*).<br>• If the data are all contained **within the manuscript and/or Supporting Information files**, enter the following: *All relevant data are within the manuscript and its Supporting Information files.*<br>• If neither of these applies but you are able to provide **details of access elsewhere**, with or without limitations, please do so. For example:<br><br>*Data cannot be shared publicly because of [XXX]. Data are available from the XXX Institutional Data Access / Ethics Committee (contact via XXX) for researchers who meet the criteria for access to confidential data.*<br><br>*The data underlying the results presented in the study are available from (include the name of the third party*	All relevant data are within the manuscript and its Supporting Information files. The corresponding author will provide the details data on a valid request if required.

and contact information or URL).<br>• This text is appropriate if the data are owned by a third party and authors do not have permission to share the data.<br><br>* typeset	
Additional data availability information:	

Manuscript

Click here to access/download;Manuscript;Manuscript_Final_IH.docx

The association of missense variant *DCLRE1B* rs3761936 with breast and cervical cancer risk - A case-control study

Sarah Jafrin[1,2], Md. Abdul Aziz[1,2,3], Md Abdul Barek[1,2,3], Md. Sharif Reza[1], Nura Ershad Naznin[1,2], Mohammad Safiqul Islam[1,2,3,*]

[1] Department of Pharmacy, Faculty of Science, Noakhali Science and Technology University, Sonapur 3814, Bangladesh

[2] Laboratory of Pharmacogenomics and Molecular Biology, Department of Pharmacy, Noakhali Science and Technology University, Noakhali 3814, Bangladesh

[3] Bangladesh Pharmacogenomics Research Network (BdPGRN), Noakhali 3814, Bangladesh

***Corresponding Author:**

Dr. Mohammad Safiqul Islam

Professor, Department of Pharmacy

Noakhali Science and Technology University

Noakhali- 3814, Bangladesh

Mobile: +8801727658650

Email: research_safiq@yahoo.com

ORCID: 0000-0003-4924-5319

The association of missense variant *DCLRE1B* rs3761936 with breast and cervical cancer risk - A case-control study

Abstract

Background: Overexpression of rs3761936 of *DCLRE1B* gene has been observed in both breast cancer and cervical cancer patients. To justify the association of this polymorphism with these cancers, we performed this case-control study.

Method: A total of 245 cancer patients and 108 healthy controls participated in the research. An efficient T-ARMS PCR method was used for genotyping.

Results: The cancer patients showed higher mutant allele frequency compared to the controls. Mutant allele carrier breast cancer patients showed significantly increased risk in four genetic models, including additive model 1 (TC vs. TT: OR=2.31, 95% CI=1.33-3.99, *p*-value=0.0028), additive model 2 (CC vs. TT: OR=3.93, 95% CI=1.36-11.38, *p*-value=0.0116), dominant model (TC+CC vs. TT: OR=2.52, 95% CI=1.50-4.25, *p*-value=0.0005), and over-dominant model (TC vs. TT+CC: OR=1.93, 95% CI=1.13-3.28, *p*-value=0.0152). The allele frequency analysis showed that mutant allele C carriers among breast cancer patients had a significantly higher risk than the wild type T allele carriers (C vs. T: OR=2.15, 95% CI=1.41-3.26, *p*-value=0.0003). Likewise, the cervical cancer patients showed significant risk in three genetic models, including additive model 1 (TC vs. TT: OR=1.80, 95% CI=1.01-3.20, *p*-value=0.0444), additive model 2 (CC vs. TT: OR=3.17, 95% CI=1.05-9.55, *p*-value=0.0403), and dominant model (TC+CC vs. TT: OR=1.98, 95% CI=1.15-3.41, *p*-value=0.0138). The mutant allele C carriers had a significantly higher risk than the wild-type T allele carriers (C vs. T: OR=1.84, 95% CI=1.19-2.85, *p*-value=0.0065).

Conclusion: *DCLRE1B* rs3761936 is strongly associated with breast cancer and cervical cancer risk in Bangladeshi women.

Keywords: *DCLRE1B*, breast cancer, cervical cancer, polymorphism, rs3761936, T-ARMS PCR

1. Background

Cancer is an incessantly evolving critical health problem that results from the attenuation of multiple somatic and germline mutations [1]. Only in 2020, about 19.3 million new cases of cancer were registered, which caused 10 million deaths worldwide [2,3]. Genetic mutations are the inevitable facts in cancer development. Next-generation sequencing (NGS) has been playing a massive role in large-scale genomic discoveries and till now has revealed tremendous information about the fundamental genomic candidates that are directly or indirectly engaged in cancer development and progression [4]. Many recent genome-wide association studies have discovered a number of somatic and germline mutations in tumor suppressor genes, such as *TP53*, *RB1*, and *APC*. Some oncogenic mutations are identified as oncogenic activators from copy number variant analysis; for example- *HER2/ERBB2* and *MYC* have been identified and potentially targeted for molecular therapy [5-8]. Intense analysis of almost 50,000 cancer genomes has been conducted to evaluate multiple genomic alterations, variations, and their impact on cancer development and progression. Most of the common variants associated with cancer have already been vastly identified and analyzed [9,10]. Interest is now growing in the rare and newer variants of the driver genes for more specific findings on cancer genomics [6,11].

The most frequently diagnosed cancers among women worldwide are breast cancer (BC) and cervical cancer (CC). The mortality rate in women with cancer is highly variable. According to 2020 statistics, breast cancer was the leading cause of death among women in 110 countries, and the mortality rate was the highest for cervical cancer patients in 36 countries [12-16]. Breast cancer caused 6,846 deaths and 12,764 new cases in Bangladesh recently, while cervical cancer, the second leading cause of female death in Bangladesh, caused 5,214 fatalities and 8,068 new cases [17]. Till now, genome-wide associatiethion studies (GWASs) have discovered thousands of disease risk-associated single-nucleotide polymorphisms (raSNPs); among them, almost 150 variants are found to be susceptible to breast cancer and cervical cancer risk [18].

DCLRE1B gene is one of the vital members of the DNA cross-link repair family that remained evolutionarily conserved. The level of expression of *DCLRE1B* gene has been found to be significantly higher in the human brain (69.0). This gene encodes an essential protein referred as ‘5' exonuclease Apollo’ (60002 Da) structured by 532 amino acids that directly interact with *TERF2* in its quaternary form and partially interact with *MUS81*, *MRE11*,

FANCD2, *HSPA2*, *HSPA8*, *HSPA14*, and *SPAG5*. The exonuclease participates in the non-homologous end-joining (NHEJ)-mediated repair where *TERF2* directs 5'-3' exonuclease to form a telomeric loop (T loop) during replication and expose the telomere end in a manner that activates the DNA repair pathways. This protein also facilitates double-strand break formation in response to DNA inter-strand cross-links. Diseases associated with *DCLRE1B* include Hoyeraal Hreidarsson Syndrome and Dyskeratosis Congenita [19-27].

The missense variant rs3761936 of *DCLRE1B* gene is located in the chr1:113907040 (GRCh38.p13) chromosomal anchor position. A missense mutation occurs when the change of a single base pair (bp) causes the substitution of a different amino acid in the resulting protein. This amino acid substitution may have no effect, or it may render the protein nonfunctional. A recent GTEx and ULCAN database showed significantly high expression of *DCLRE1B* mRNA in mammary breast tissue (**Figure 1**, **Figure 2**). The UALCAN database also revealed the presence of significantly high expression of *DCLRE1B* mRNA in cervical cancer tissues compared to healthy tissues, which indicates a vital role of the *DCLRE1B* gene in the development of cervical cancer. As missense mutations tend to modify amino acid sequences, there remains a possibility of producing inactive mRNA or hyperactive mRNA. In both cases, normal expression of mRNA would not be observed if missense mutation is present. This low- or over-expression of mRNA may produce inactive or modified proteins that are ultimately associated with various diseases or conditions. In the case of rs3761936 of *DCLRE1B* gene, this missense mutation upregulates the mRNA expression in breast cancer and cervical cancer patients. There is a significant possibility that this specific polymorphism might play a potential role in breast and cervical cancer development [28-30].

Based on the prior studies, we chose the rare genetic variant of the *DCLRE1B* gene, a bi-allelic polymorphism rs3761936, that may influence breast cancer and cervical cancer development. To the best of our knowledge, no individual study has been performed on this polymorphism in any population except the GWAS study. Therefore, the aim of our present study is to evaluate the risk association of breast cancer and cervical cancer development with *DCLRE1B* rs3761936 polymorphism in Bangladeshi women.

2. Method and Materials

2.1. Study Setting

This case-control study was performed at the Laboratory of Molecular Biology and Pharmacogenomics of Noakhali Science and Technology University. The 1964 Helsinki Declaration and its later revisions, or equivalent ethical standards, were followed in all procedures conducted in research involving human subjects. The Ethical Review Board of the National Institute of Cancer Research and Hospital approved the study protocol (for breast cancer: NICRH/Ethics/2019/446 and for cervical cancer: NICRH/Ethics/2019/447). Patients' informed consent was obtained in advance. Three groups of participants were selected for the study. Two groups consisting of 135 women with breast cancer and 110 women with cervical cancer (aged 25-70 years) were recruited as cases from the National Institute of Cancer Research Hospital (NICRH), and 108 healthy volunteers (aged 21-74 years) from the volunteers who came to the hospital for their routine check-ups in NICRH were selected as controls. The blood samples were collected from July 1, 2019, to March 30, 2020. All the participants submitted their written informed consent prior to blood collection. Participants were all legally adults (above the age of 18) and genetically unrelated to others. The degree of heterogeneity between the demographic features of cases and controls was kept to a minimum. A standard questionnaire concerning disease history, as well as a genetic record of breast cancer in the family tree, were used to determine the patient's medical history. All the patients were also clear of other serious illnesses such as lung, renal, and liver disease. The information of the recruited control population revealed no evidence of any personal history of cancer or other long-term illnesses. The seventh edition of the American Joint Committee on Cancer (AJCC) tumor-node-metastasis (TNM) staging system was used to classify breast cancer and cervical cancer.

2.2. Sample collection, storage, and DNA extraction

For sampling and DNA extraction, 3 ml of blood was drawn from each patient and control participant, placed in sterile tubes with EDTA-Na_2, and stored at -80°C until DNA extraction. Following the extraction procedure described by I[illegible]m et al., genomic DNA was isolated [31].

2.3. Genotyping and Screening

Utilizing all individuals' extracted DNA for genotyping, we next used T-ARMS PCR to amplify the target DNA fragments. Following the procedure described by previous studies from our lab [32, 33], the SNP was genotyped. Licensed software was used to create the

primers. To find the desired polymorphism, agarose gel electrophoresis was used to examine amplified DNA fragments (**Table 1**).

2.4. Method validation for selecting annealing temperature

The annealing temperature of a PCR is a crucial parameter that depends on the melting point of the primers. The temperature must be at least 5℃ lower than the primers' melting point (Tm). We estimated Tm by utilizing Primer Blast software (**Table S1**). The annealing temperature may deviate from the expected value because of buffer or salt content. In order to execute gradient PCR, we chose 6 different annealing temperatures between 59.5℃ and 62℃ and used our customized master mix formula for the procedure. We used two identical DNA samples at various temperatures to figure out the ideal temperature. After PCR, we used gel electrophoresis to observe the DNA bands of an individual allele. At 61℃, we detected 344 bp, 220 bp, and 180 bp size fragments, and their appearance was more pronounced than at the other temperatures. 61℃ was therefore chosen as our preferred annealing temperature. We also reanalyzed 20% heterozygous genotypes to confirm the reliability of the genotyping.

2.5. Statistical Analysis

Two-sided unpaired t-tests and the chi-square test (χ^2) were used to examine the distributions of the demographic factors between the cases and the controls. The percentages of genotype and allelic frequencies were reported. To determine the strength of the link between this SNP and Bangladeshi breast cancer and cervical cancer patients, the odds ratio (OR) and their 95% confidence intervals (CI) were calculated by SPSS software package version 24.0 (SPSS, Inc., Chicago, IL). Results were deemed significant when the *p*-value was less than 0.05 and non-significant when the *p*-value was more than 0.05.

3. Results

3.1. Cases and controls characteristics

The distributions of demographic characteristics of 135 breast cancer patients, 110 cervical cancer patients, and 108 controls are summarized in **Table 2**. The age range of cancer patients was 25-70 and 21-74 for the control population. The average age for patients was 45.65 years and for controls was 40.37 years. The average body mass index (BMI) of patients was 29.39 kg/m^2 and of controls was 22.30 kg/m^2. This table also showed other characteristics,

including- smoking habit, marital status, menstrual commencement, menopause, childbirth frequency, and breastfeeding status. Most of the patients (76.70%) first showed their symptoms above 40 years of age. 70.10% of patients had a history of taking contraceptive pills. Among the pill users, 52.13% of patients took pills for more than 5 years, and 47.87% of patients continued for less than 5 years, whereas 23 patients had a family history of different cancers.

Tables 3 and **4** show histopathological data of the BC and CC patients, respectively. According to BC patients' medical history, 62.96% of patients had invasive duct cell carcinoma, and 25.19% had infiltrating duct cell carcinoma, 3.70% patients had duct cell carcinoma, 2.22% had metastatic duct cell carcinoma, 1.48% had atypical ductal hyperplasia and intraductal carcinoma, and 2.96% had triple-negative breast cancer. TNM staging system reported the tumor size of the individuals. Nodal status data reported that most of the patients had N1 type tumor. We also included tumor grade, ER, PR, and HER2 status of breast cancer patients.

Of the 110 CC patients, 74.55% suffered from squamous cell carcinoma and 25.45% suffered from adenocarcinoma. According to tumor stage data, the majority of the patients had IIB type tumor. Cancer grading percentage was- grade 1 (26.36%), grade 2 (64.55%), and grade 3 (9.09%). Based on the tumor size analysis, 52.72%, 36.36%, 8.18%, and 2.72% of patients had T1, T2, T3, and T4 size, respectively. 84.61% of patients showed negative lymph node status as per our database. Nodal status was also included for the individuals and 52.63% had nodal status. According to distant metastasis data, patients were grouped into three groups- Mx (74.76%), M0 (22.33%), and MI (2.91%).

3.2. Genotype and allele frequencies distribution

We performed the Hardy-Weinberg equilibrium (HWE) test for both patients and controls. Breast cancer patients had comparatively higher mutant homozygote and mutant allele frequency than in the control population. Based on our analysis of *DCLRE1B* rs3761936 polymorphism in the BC patients, genotypic frequency distribution was consistent with the HWE test (χ^2=0.02, *p-value* = 0.891). The frequency of the reference allele T was 80.09%, and mutant allele C was 19.91% in the control population (χ^2=0.19, *p-value* = 0.664). Cervical cancer patients showed similar genotypic distribution to breast cancer patients. The frequency of the reference homozygote was higher in the control population than in the cervical cancer population. Genotypic frequency distribution was consistent with the HWE test (χ^2=0.27, *p-value* = 0.601). Noticeable differences between the genotypic frequencies of the patients and

controls indicate that *DCLRE1B* rs3761936 polymorphism might be associated with an increased risk of breast cancer (**Table S2**).

3.3. Association between breast cancer and rs3761936 polymorphism

We used six genetic models to quantify the risk of *DCLRE1B* rs3761936 polymorphism on breast cancer patients (**Table 5**). The risk was estimated by the odds ratios of each genetic model, and the control population was considered as a reference. For breast cancer patients, additive model 1 showed 2.31-fold significantly increased risk of cancer among the heterozygote TC carriers (TC vs. TT: OR=2.31, 95%CI=1.33-3.99, *p*-value=0.0028). Additive model 2 showed 3.93-fold increased risk among the mutant homozygote CC carriers against reference homozygote carriers (CC vs. TT: OR=3.93, 95%CI=1.36-11.38, *p*-value=0.0116). According to the dominant model, 2.52-fold significant risk was identified in the breast cancer patients against the control population (TC+CC vs. TT: OR=2.52, 95%CI=1.50-4.25, *p*-value=0.0005). The recessive model also showed 2.77-fold increased risk in cancer patients, although the result was not statistically significant (*p*-value=0.0545). The over-dominant model showed 1.93-fold significant risk in breast cancer patients (TC vs. TT+CC: OR=1.93, 95%CI=1.13-3.28, *p*-value=0.0152). The allele frequency analysis showed that mutant allele C carriers among breast cancer patients had a significantly higher risk than the wild type T allele carriers (C vs. T: OR=2.15, 95%CI=1.41-3.26, *p*-value=0.0003).

3.4.Association between cervical cancer and rs3761936 polymorphism

For cervical cancer patients, additive model 1 showed 1.80-fold significantly increased risk of cancer among the heterozygote TC carrier (TC vs. TT: OR=1.80, 95%CI=1.01-3.20, *p*-value=0.0444). Additive model 2 showed 3.17-fold increased risk among the mutant homozygote CC carriers against reference homozygote carriers (CC vs. TT: OR=3.17, 95%CI=1.05-9.55, *p*-value=0.0403). According to the dominant model, 1.98-fold significant elevated risk was identified in the cervical cancer patients against the control population (TC+CC vs. TT: OR=1.98, 95%CI=1.15-3.41, *p*-value=0.0138). Both the recessive model and over-dominant models also showed increased risk in the cervical cancer patients against the reference population, although the results were not statistically significant (CC vs. TT+TC: OR=2.52, 95%CI=0.86-7.42, *p* -value=0.0929; TC vs. TT+CC: OR=1.57, 95%CI=0.9-2.75, *p* -value=0.1118). The allele frequency analysis showed that mutant allele C carriers among cervical cancer patients had a significantly higher risk than the wild type T allele carriers (C vs. T: OR=1.84, 95%CI=1.19-2.85, *p*-value=0.0065) (**Table 6**).

4. Discussion

A key variable in the emergence of cancer is genetic polymorphism. Extensive research has been ongoing to find more precise and novel causes of cancer genetic vulnerability. Until now, more than 10,000 variants have been identified as susceptible candidates, although population-based studies sometimes show highly variable outcomes. Scientists are now attempting to identify rare variants that might have population-specific susceptibility to cancer. Among all types of cancers, breast cancer and cervical cancer are the most dominant cancers in women, especially underdeveloped countries suffer a lot from these two types of cancers due to the lack of screening and early detection or lack of awareness. In Bangladesh, female breast cancer and cervical cancer are the most common types of cancers among women, causing 12,060 deaths in 2018 [17, 34-36]. Early detection and prevention can reduce the prevalence, incidence and mortality of the disease, but genetic susceptibility studies can play a dominant role in designing personalized medications that could prevent the occurrence of cancers due to genetic causes. Although rare variant susceptibility studies can alter the disease scenario, there are very few genetic susceptibility studies on breast cancer and cervical cancer in Bangladesh.

DCLRE1B gene plays a vital role in telomere maintenance and protection during the S-phase of DNA replication. DCLRE1B protein is highly expressed in the telomere site of the chromosomes and interacts with TERF2, and involved in prophase checkpoint and prevents intra-strand DNA cross-linking by exonuclease activities. The rs3761936 of *DCLRE1B* gene is a missense variant that miscodes amino acid sequences. The altered amino acid sequence produces a different protein that might be highly susceptible to hamper DNA cross-link repair leading to the development of most cancers [19-21, 26, 27].

Although the rs3761936 polymorphism of *DCLRE1B* gene might have a high susceptibility to developing breast cancer and cervical cancer, no specific case-control studies were conducted to evaluate the association. Only a few genetic databases showed that protein expression with the polymorphic allele of *DCLRE1B* gene was over-expressed in both breast invasive cancer cells and cervical carcinoma cells [28-30]. In a previous study, Dong et al. conducted a huge cancer association study with 344 genetic variants, of which rs3761936 polymorphism of *DCLRE1B* gene was found to be significantly associated with glioma, although the polymorphism showed protective association (OR=0.36) [34]. Another whole

genome association study on rheumatoid arthritis (RA) patients of New Zealand and the United Kingdom [37] showed that the minor allele C frequency of rs3761936 SNP was significantly higher in the RA patients of both regions in comparison to the control population (*p*-value=0.001). Another study on glioma patients also found that the minor allele frequency was significantly higher in patients than in control, and this SNP showed a lower risk for glioma [38]. GEPIA (Gene Expression Profiling Interactive Analysis) and OncoDB databases reported overexpressed *DCLRE1B* gene in both breast cancer and cervical cancer patients [39,40] as shown in **Figure 3** and **Figure 4**. Although the evidence was somehow confusing and contradictory, we aimed to evaluate the risk association of *DCLRE1B* rs3761936 polymorphism in Bangladeshi breast cancer and cervical cancer patients.

In this case-control study, among the breast cancer patients, 45.93% showed heterozygote TC and 11.85% showed mutant homozygote CC. In the case of allele frequency, reference allele T frequency was 65.18% and mutant allele C frequency was 34.81%. Among the cervical cancer patients, 40.91% showed heterozygote TC and 10.91% showed mutant homozygote CC. In case of allele frequency, reference allele T frequency was 68.64% and mutant allele C frequency was 31.36%. Minor allele frequency was significantly higher in both the breast cancer and cervical cancer patients than in the control population. In genetic model analysis of breast cancer patients, additive model 1 showed 2.31-fold significantly increased risk among the heterozygote TC carriers (*p*-value=0.0028). 3.93-fold increased risk was identified in additive model 2 among the mutant homozygote CC carriers (*p*-value=0.0116). The dominant model showed 2.52-fold significant risk in the breast cancer patients against the control population (*p*-value=0.0005). The recessive model also showed increased risk, but the result was not statistically significant (*p*-value=0.0545). The over-dominant model also showed 1.93-fold significant risk in breast cancer patients (*p*-value=0.0152). The mutant allele C carriers in the breast cancer patients had a significantly higher risk than the wild-type T allele carriers (*p*-value=0.0003).

For cervical cancer patients, similar results were observed. Additive model 1 showed 1.80-fold significantly increased risk in the heterozygote TC carrier (*p*-value=0.0444). 3.17-fold increased risk was identified in additive model 2 among the mutant homozygote CC carriers (*p*-value=0.0403). The dominant model showed 1.98-fold significant risk in the cervical cancer patients against the control population (*p*-value=0.0138). Both the recessive model and over-dominant models showed increased risk in the cervical cancer patients against the reference population, although the results were not statistically significant (*p*-value>0.05).

The allele frequency analysis showed that mutant allele C carriers had a significantly higher risk than the wild-type T allele carriers (*p*-value=0.0065).

Although this is the first case-control study of *DCLRE1B* rs3761936 polymorphism with the risk of breast and cervical cancer, there were some limitations that should be mentioned. The sample size of the patients was limited. For genetic association studies, the larger the sample size, the stronger the consistency and stability of the results. Some of our results were not statistically significant may be due to the smaller sample size. Apart from the smaller sample size, we were able to report consistency with the HWE constant. Except for the mentioned limitations, our present case-control study confirmed that *DCLRE1B* rs3761936 polymorphism has a strong association with breast cancer and cervical cancer risk in Bangladeshi women.

5. Conclusion

DCLRE1B rs3761936 polymorphism is strongly associated with a significantly increased risk of breast cancer and cervical cancer in Bangladeshi women. Our study showed robust evidence that can be considered valuable for precise diagnosis of breast cancer and cervical cancer patients. However, a single study cannot establish firm evidence as genetic variations among different ethnicities are highly noticeable. So, further studies with larger samples on the other ethnic population should be conducted to confirm and re-evaluate our findings.

Acknowledgments

The authors would like to thank the Department of Pharmacy, Noakhali Science and Technology University, for their assistance and support during the project. Cordial thanks to the National Institute of Cancer Research and Hospital (NICRH), Dhaka, for their valuable contribution.

Funding

The Research Cell, Noakhali Science and Technology University, Noakhali-3814, Bangladesh, funded this study partially (NSTU/RC/20/C-86), and no other public or private logistic funding was provided.

Conflicts of interest

The authors declare no conflicts of interest.

Availability of data and materials

All the related data and materials will be accessible upon request to the corresponding author.

References

1. Nakagawa H, Fujita M. Whole genome sequencing analysis for cancer genomics and precision medicine. *Cancer Sci*. 109(3), 513–22 (2018).

2. Sung H, Ferlay J, Siegel RL et al. Global cancer statistics 2020: GLOBOCAN estimates of incidence and mortality worldwide for 36 cancers in 185 countries. *CA Cancer J Clin* 0(0), 1–41 (2021).

3. Aziz MA, Jafrin S, Islam MS. Human TERT promoter polymorphism rs2853669 is associated with cancers: an updated meta-analysis. *Human Cell* 34, 1066–1081 (2021).

4. Berger MF, Mardis ER. The emerging clinical relevance of genomics in cancer medicine. *Nat Rev Clin Oncol* 15(6), 353–65 (2018).

5. Stratton MR, Campbell PJ, Futreal PA. The cancer genome. *Nature* 458(7239), 719–24 (2009).

6. Garraway LA LE. Lessons from the cancer genome. *Cell* 153, 17–37 (2013).

7. Kinzler KW, B. V. Lessons from hereditary colorectal cancer. *Cell* 87, 159–70 (1996).

8. King CR, M K, SA. A. Amplification of a novel v-erbB related gene in human mammary carcinoma. *Science* 229, 974–6, (1985).

9. Vogelstein B, Papadopoulos N, Velculescu VE, Zhou S, Diaz LA Jr KK. Cancer genome landscapes. *Science* 339, 1546–58 (2013).

10. Lawrence MS, Stojanov P, Mermel CH et al.. Discovery and saturation analysis of cancer genes across 21 tumor types. *Nature* 505, 495–501 (2014).

11. Leiserson MDM, Vandin F, Wu H-T et al. Pan-cancer network analysis identifies combinations of rare somatic mutations across pathways and protein complexes. *Nat Genet* 47(2), 106–14 (2015).

12. Nahar Z, Jafrin S, Aziz MA, Islam MS. Link of IL-1β rs16944 polymorphism with breast, cervical and ovarian cancer: A systematic review and meta-analysis. *Gene Reports* 26, 101539 (2022).

13. Aziz MA, Jafrin S, Islam MS, Kabir Y. Interleukins in the Development and Progression of Breast Cancer. In: *Interdisciplinary Cancer Research*. Springer, Cham (2022).

14. Jafrin S, Aziz MA, Islam MS. Association between *TP73 G4C14-A4T14* polymorphism and different cancer types: an updated meta-analysis of 55 case-control studies. *J Int Med Res* 50(10), 3000605221133173 (2022).

15. Aziz MA, Islam MS. MAP3K1 rs889312 polymorphism and cancer prognosis: A systematic review and meta-analysis. *Cancer Rep (Hoboken)* 6(1), e1773 (2023).

16. Aziz MA, Uddin MS, Millat MS, Islam MS. Vascular endothelial growth factor A (VEGFA) promoter rs2010963 polymorphism and cancer risk: An updated meta-analysis and trial sequential analysis. *Meta Gene* 31, 101017 (2022).

17. Bray F, Ferlay J, Soerjomataram I et al. Global cancer statistics 2018: GLOBOCAN estimates of incidence and mortality worldwide for 36 cancers in 185 countries. *CA Cancer J Clin* 68(6), 394–424 (2018).

18. Guo X, Lin W, Bao J et al. A Comprehensive cis-eQTL Analysis Revealed Target Genes in Breast Cancer Susceptibility Loci Identified in Genome-wide Association Studies. *Am J Hum Genet* 102(5), 890–903 (2018).

19. Ishiai M, Kimura M, Namikoshi K et al. DNA cross-link repair protein SNM1A interacts with PIAS1 in nuclear focus formation. *Mol. Cell. Biol* 24, 10733-10741 (2004).

20. Demuth I, Digweed M, Concannon P. Human SNM1B is required for normal cellular response to both DNA interstrand crosslink-inducing agents and ionizing radiation. *Oncogene* 23, 8611-8618 (2004).

21. van Overbeek M, de Lange T. Apollo, an Artemis-related nuclease, interacts with TRF2 and protects human telomeres in S phase. *Curr. Biol* 16, 1295-1302 (2006).

22. Lenain C, Bauwens S, Amiard S et al. The Apollo 5' exonuclease functions together with TRF2 to protect telomeres from DNA repair. *Curr. Biol* 16, 1303-1310 (2006)

23. Demuth I, Bradshaw PS, Lindner A et al. Endogenous hSNM1B/Apollo interacts with TRF2 and stimulates ATM in response to ionizing radiation. *DNA Repair* 7, 1192-1201 (2008).

24. Bae JB, Mukhopadhyay SS, Liu L et al. Snm1B/Apollo mediates replication fork collapse and S Phase checkpoint activation in response to DNA interstrand cross-links. *Oncogene* 27, 5045-5056 (2008).

25. Liu L, Akhter S, Bae JB et al. SNM1B/Apollo interacts with astrin and is required for the prophase cell cycle checkpoint. *Cell Cycle* 8, 628-638 (2009).

26. Anders M, Mattow J, Digweed M, Demuth I. Evidence for hSNM1B/Apollo functioning in the HSP70 mediated DNA damage response. *Cell Cycle* 8, 1725-1732 (2009).

27. Ye J, Lenain C, Bauwens S et al. TRF2 and Apollo cooperate with topoisomerase 2alpha to protect human telomeres from replicative damage. *Cell* 142, 230-242 (2010).

28.GTEx Project. GTEx Analysis Release V6p .2017.p.2.z https://gtexportal.org/home/eqtls/tissue?tissueName=Breast_Mammary_Tissue

29.ULCANdatabase.http://ualcan.path.uab.edu/cgibin/TCGAExResultNew2.pl?genenam =DCLRE1B&ctype=BRCA

30.ULCANdatabase.http://ualcan.path.uab.edu/cgibin/TCGAExResultNew2.pl?genenam =DCLRE1B&ctype=CESC

31. Islam MS, Ahmed MU, Sayeed MS et al. Lung cancer risk in relation to nicotinic acetylcholine receptor, CYP2A6 and CYP1A1 genotypes in the Bangladeshi population. *Clin Chim Acta* 416, 11-19 (2013).

32. Aziz MA, Akter T, Hussain MS et al. Association of rs363598 and rs360932 polymorphisms with autism spectrum disorder in the Bangladeshi children. *Meta Gene* 25, 100733 (2020).

33. Hasan ME, Matin M, Haque ME, et al. Polymorphic variants INSIG2 rs6726538, HLA-DRB1 rs9272143, and GCNT1P5 rs7780883 contribute to the susceptibility of cervical cancer in the Bangladeshi women. *Cancer Med* 10(5), 1829-1838 (2021).

34. Dong LM, Potter JD, White E et al. Genetic susceptibility to cancer: The role of polymorphisms in candidate genes. *JAMA - J Am Med Assoc* 299(20), 2423–36 (2008).

35. Jafrin S, Aziz MA, Islam MS. Impact of interleukin-32 germline rs28372698 and intronic rs12934561 polymorphisms on cancer development: A systematic review and meta-analysis. *Int Immunopharmacol* 99, 107964 (2021).

36. Barek MA, Basher MA, Aziz MA et al. Assessment of the association of CYP1A1 gene polymorphisms with the susceptibility of cervical cancer: A case-control study and meta-analysis. *Heliyon* 9(7), e17712 (2023).

37. Steer S, Abkevich V, Gutin A et al. Genomic DNA pooling for whole-genome association scans in complex disease: Empirical demonstration of efficacy in rheumatoid arthritis. *Genes Immun* 8(1), 57–68 (2007).

38. Bethke L, Webb E, Murray A et al. Comprehensive analysis of the role of DNA repair gene polymorphisms on risk of glioma. *Hum Mol Genet* 17(6), 800–5 (2008).

39. GEPIA (Gene Expression Profiling Interactive Analysis). Available from: http://gepia2.cancer-pku.cn/#analysis

40. OncoDB DATABASE. https://oncodb.org/genomic_profile_express_non_virus.html.

Figure legends

Figure 1: GTEx database showing high expression of DCLRE1B mRNA in breast cancer vs. normal mammary tissue at the presence of rs3761936 polymorphism.

Figure 2: UALCAN database showing significantly high expression of *DCLRE1B* mRNA in both cervical cancer and breast invasive carcinoma tissue compared to healthy tissues.

Figure 3: GEPIA2 database on the genetic expression of *DCLRE1B* gene in breast cancer and cervical cancer patients.

Figure 4: OncoDB report on the genetic expression of *DCLRE1B* gene in breast cancer and cervical cancer patients .

Table 1: PCR condition to amplify the *DCLRE1B* rs3761936 and the respective length of PCR products

SNP	*PCR conditions*	*No. of cycles*	*Size of PCR products (bp)*	*Genotypes*
DCLRE1B rs3761936	95℃ for 5 minutes **Initial denaturation**			
	95℃ for 1 minute **Denaturation**	35 cycles	NH: 220, 344 bp	TT
	61℃ for 45 seconds **Annealing**		HE: 180, 220, 344 bp	TC
	72℃ for 1 minute **Extension**		MH:180, 344 bp	CC
	72℃ for 10 minutes **Final extension**			

* NH- Normal Homozygote, HE- Heterozygote, MH- Mutant Homozygote

Table 2: Distribution of demographic variables of cancer patients and controls

Variables	***Cases n=245 (%)***	***Controls n=108 (%)***
Age (years)		
Minimum Age	25	21
Maximum Age	70	74
Average Age	45.65	40.37
BMI (kg/m²)		
Average	29.39	22.30
Marital Status		
Married	238 (97.14)	93 (86.11)
Unmarried	7 (2.86)	15 (13.89)
Region of Patient		
Urban	59.09	56.77
Rural	40.91	43.23
Menstruation Cycle Starting Age		
≤13	68.18	28.82
>13	31.82	71.18
Menopausal Age	n = 150	n=50
≤45	80.12	24.22
>45	19.88	75.78
First Child Conceived Age		
≤18	75.45	60.49
>18	24.55	39.51
Breastfeeding period		
<2	14.29	28.34
≥2	85.71	71.66
Commencement of Cancer Symptoms		
≤40	23.30	NA
>40	76.70	NA
History of Taking Contraceptive Pills	n=167	n=70
Yes	68.16	64.81
No	31.84	35.19
Smoking History	None	None
Family History of Cancer	20.82	None

*NA- Not Applicable

Table 3: Distribution of clinicopathological features of breast cancer patients

Variables	*Cases, n=135 (%)*
Histological Types of Breast Cancer	
Atypical ductal hyperplasia	2 (1.48)
Duct cell carcinoma	5 (3.70)
Infiltrating duct cell carcinoma	34 (25.19)
Intraductal carcinoma	2 (1.48)
Invasive duct cell carcinoma	85 (62.96)
Metastatic duct cell carcinoma	3 (2.22)
Triple negative breast cancer	4 (2.96)
TNM Staging System	
Tumor size	
Tx	0
Tis	0
T0	27 (20.00)
T1	45 (33.33)
T2	34 (25.19)
T3	14 (10.37)
T4	15 (11.11)
Nodal status	
Nx	25 (18.52)
N0	19 (14.07)
N1	59 (43.70)
N2	15 (11.11)
N3	17 (12.59)
Distant metastasis	
M0	102 (75.56)
M1	33 (24.44)
Grade of Breast Cancer	
I	23 (17.03)
II	89 (65.93)
III	23 (17.03)
ER Status	
ER (+)	56 (41.48)
ER (-)	79 (58.52)
PR Status	
PR (+)	57 (42.22)
PR (-)	78 (57.78)
HER2 Status	
HER2 (+)	60 (44.44)
HER2 (-)	75 (55.56)

Table 4: Clinicopathological features of cervical cancer patients

Variables	***Cases (%)***
Type of Cancer	
Squamous cell carcinoma	82 (74.55)
Adenocarcinoma	28 (25.45)
Tumor Stage (N = 103)	
I	9 (8.74)
IIB	52 (50.49)
IIIA	3 (2.91)
IIIB	36 (34.95)
IVA	3 (2.91)
Grade of Cancer	
Grade 1	29 (26.36)
Grade 2	71 (64.55)
Grade 3	10 (9.09)
Tumor Size	
T1	58 (52.72)
T2	40 (36.36)
T3	9 (8.18)
T4	3 (2.72)
Lymph Node Status (N = 104)	
Negative (-)	88 (84.61)
Positive (+)	18 (17.30)
Nodal Status	
N1	10 (52.63)
N2	8 (42.11)
N3	1 (5.26)
Distant Metastasis	
Mx	77 (74.76)
M0	23 (22.33)
M1	3 (2.91)

Table 5: Quantitative risk analysis of *DCLRE1B* rs3761936 polymorphism on breast cancer patients

SNP	Model	Genotype /Allele	Cases (%)	Controls (%)	OR	95% CI	*p*-value
DCLRE1B rs3761936	Additive model 1 (TC vs. TT)	TT	57 (42.22)	70 (64.81)	1		
		TC	62 (45.93)	33 (30.56)	2.31	1.33-3.99	**0.0028**
	Additive model 2 (CC vs. TT)	CC	16 (11.85)	5 (4.63)	3.93	1.36-11.38	**0.0116**
	Dominant model (TC+CC vs. TT)	TT	57 (42.22)	70 (64.81)	1		
		TC+CC	78 (57.78)	38 (35.19)	2.52	1.50-4.25	**0.0005**
	Recessive model (CC vs. TT+TC)	TT+TC	119 (88.15)	103 (95.37)	1		
		CC	16 (11.85)	5 (4.63)	2.77	0.98-7.82	0.0545
	Over-dominant model (TC vs TT+CC)	TT+CC	73 (54.07)	75 (69.44)	1		
		TC	62 (45.93)	33 (30.56)	1.93	1.13-3.28	**0.0152**
	Allele model	T	176 (65.18)	173 (80.09)	1		
		C	94 (34.81)	43 (19.91)	2.15	1.41-3.26	**0.0003**

*OR- Odd Ratio, Bold *p*-values are significant

Table 6: Quantitative risk analysis of *DCLRE1B* rs3761936 polymorphism on cervical cancer patients

SNP	Model	Genotype /Allele	Cases (%)	Controls (%)	OR	95% CI	*p*-value
DCLRE1B rs3761936	Additive model 1 (TC vs. TT)	TT	53 (48.18)	70 (64.81)	1		
		TC	45 (40.91)	33 (30.56)	1.80	1.01-3.20	**0.0444**
	Additive model 2 (CC vs. TT)	CC	12 (10.91)	5 (4.63)	3.17	1.05-9.55	**0.0403**
	Dominant model (TC+CC vs. TT)	TT	53 (48.18)	70 (64.81)	1		
		TC+CC	57 (51.82)	38 (35.19)	1.98	1.15-3.41	**0.0138**
	Recessive model (CC vs. TT+TC)	TT+TC	98 (89.09)	103 (95.37)	1		
		CC	12 (10.91)	5 (4.63)	2.52	0.86-7.42	0.0929
	Over-dominant model (TC vs TT+CC)	TT+CC	65 (59.09)	75 (69.44)	1		
		TC	45 (40.91)	33 (30.56)	1.57	0.9-2.75	0.1118
	Allele model	T	151 (68.64)	173 (80.09)	1		
		C	69 (31.36)	43 (19.91)	1.84	1.19-2.85	**0.0065**

*OR- Odd Ratio, Bold *p*-values are significant

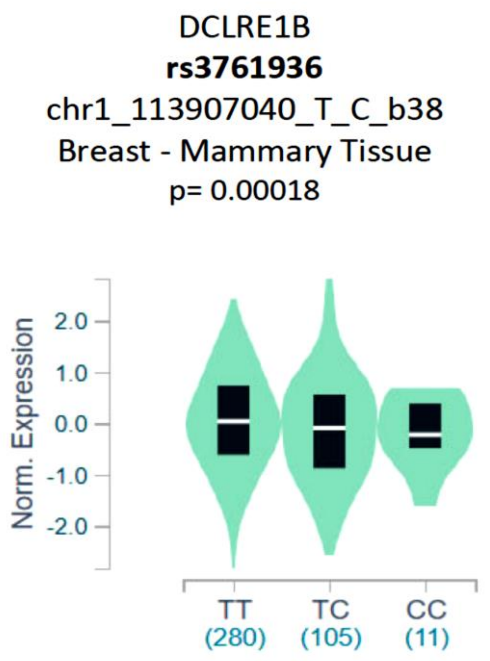

Figure 1: GTEx database showing high expression of *DCLRE1B* mRNA in breast cancer vs. normal mammary tissue at the presence of rs3761936 polymorphism [22].

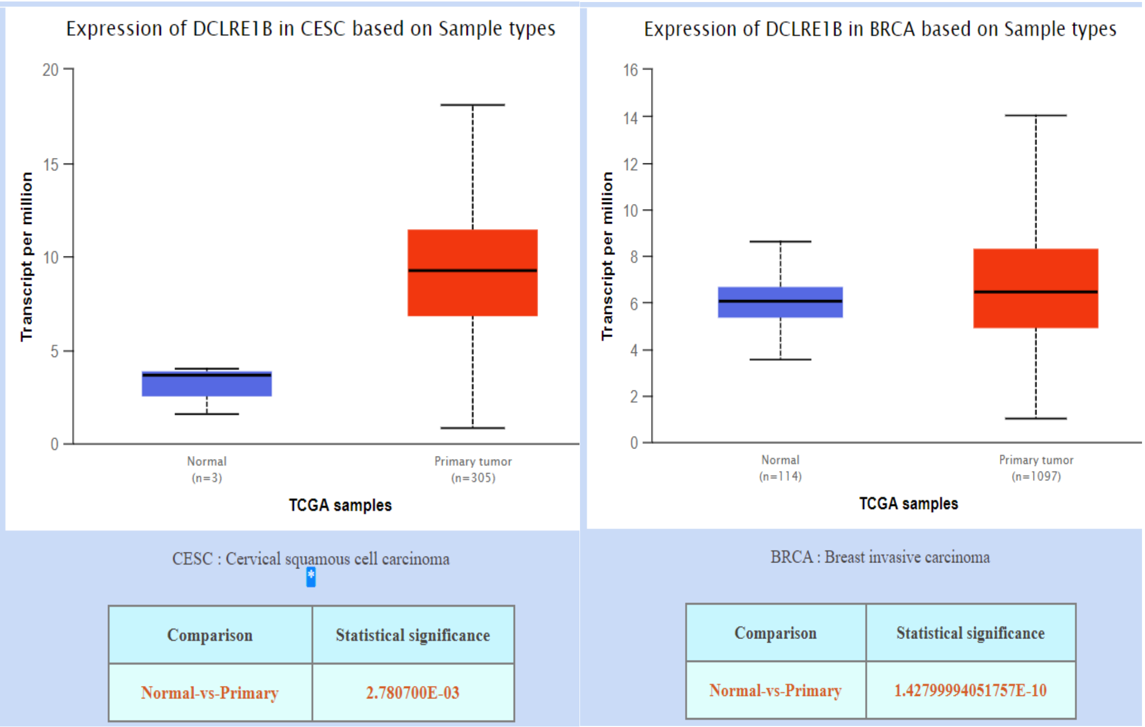

Figure 2: UALCAN database showing significantly high expression of *DCLRE1B* mRNA in both cervical cancer and breast invasive carcinoma tissue compared to healthy tissues [23, 24].

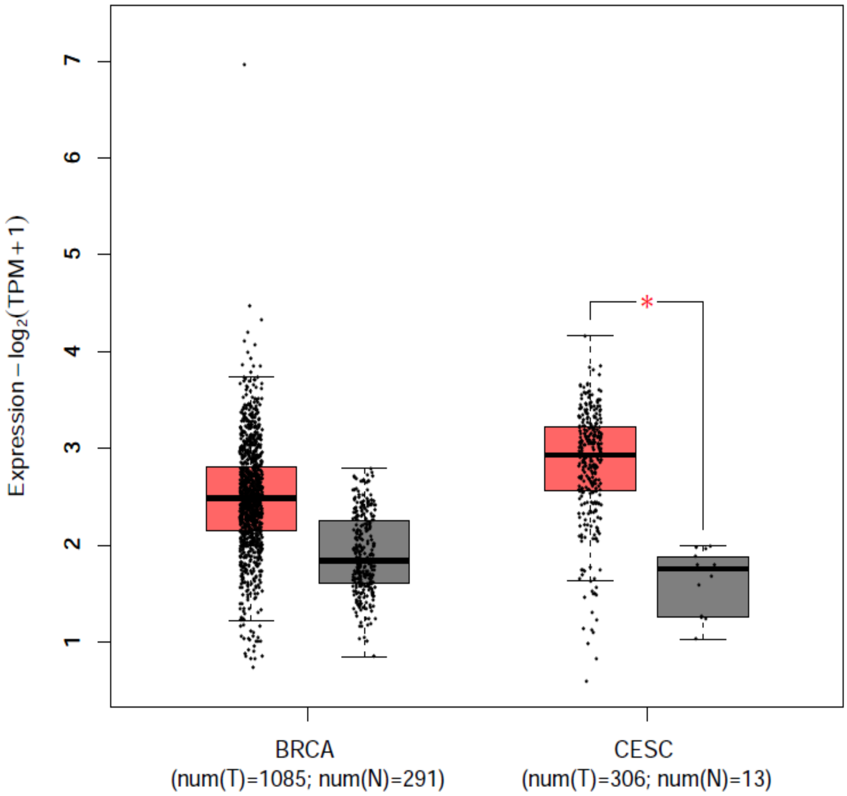

Figure 3: GEPIA2 database on the genetic expression of *DCLRE1B* gene in breast cancer and cervical cancer patients [29].

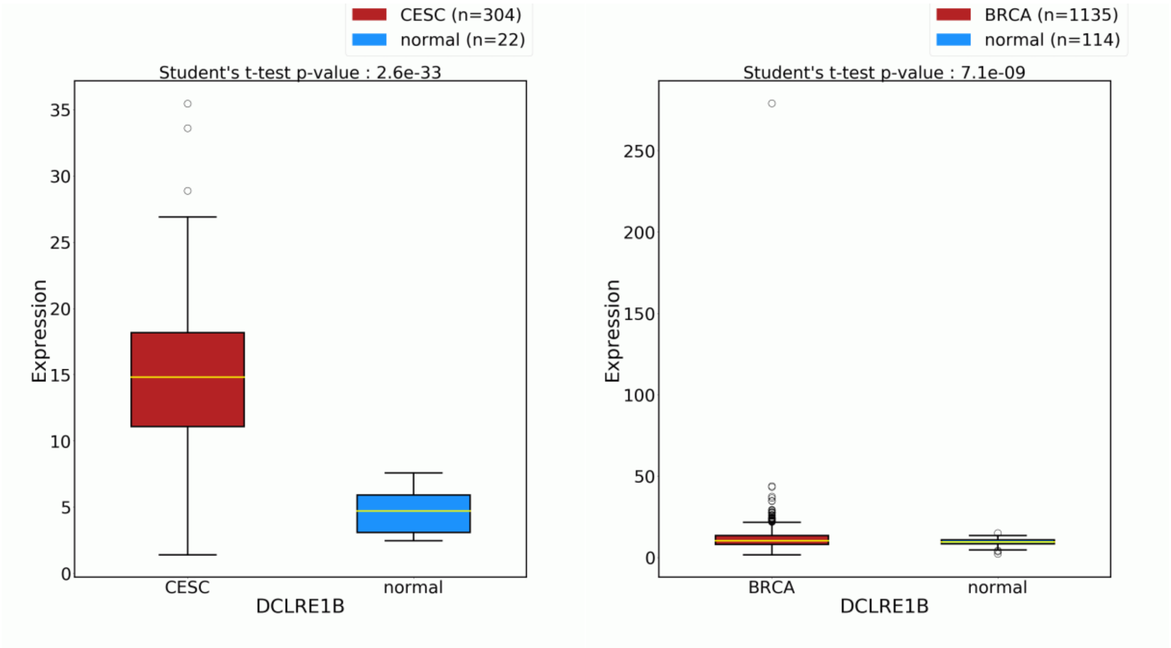

Figure 4: OncoDB report on the genetic expression of *DCLRE1B* gene in breast cancer and cervical cancer patients [30].

Click here to access/download
Supporting Information
Supplementary Tables.docx
