# Supplementary material for: Association of missense variant DCLRE1B rs3761936 with breast and cervical cancer risk—A case-control study
Source: PLoS One. 2025 Sep 8;20(9):e0331492. doi: 10.1371/journal.pone.0331492 (PMC12416678; doi:10.1371/journal.pone.0331492)
Supplement: Attachment — Submitted filename: Response.docx [file pone.0331492.s006.docx]

We are thankful to the editor and the reviewers for their crucial comments on our manuscript. We hope these comments will help us improve it.

| Required by | Reviewer’s comment | Author’s input |
| --- | --- | --- |
|  |  |  |
| Journal Academic Editor | 1.  Please ensure that your manuscript meets PLOS ONE's style requirements, including those for file naming. | 1. All the requirements are fulfilled as per journal requirements. |
|  | 2. Consider depositing your raw data in a repository to ensure your work is read, appreciated and cited by the largest possible audience. You’ll also earn an Accessible Data icon on your published paper if you deposit your data in any participating repository. | 2. This suggestion is crucial. We have not deposited these data in any repository, but we added our data as an EXCEL file as supporting materials. |
|  | 3. Please state what role the funders took in the study. If the funders had no role, please state: "The funders had no role in study design, data collection and analysis, decision to publish, or preparation of the manuscript." If this statement is not correct you must amend it as needed.  Please include this amended Role of Funder statement in your cover letter; we will change the online submission form on your behalf. | 3. The statement is included in the Funding information file. |
|  | 4. Please note that funding information should not appear in the Acknowledgments section or other areas of your manuscript. We will only publish funding information present in the Funding Statement section of the online submission form. Please remove any funding-related text from the manuscript. | 4. Removed from the manuscript and saved in a ‘financial information’ named file. |
|  | 5. In this instance it seems there may be acceptable restrictions in place that prevent the public sharing of your minimal data. However, in line with our goal of ensuring long-term data availability to all interested researchers, PLOS’ Data Policy states that authors cannot be the sole named individuals responsible for ensuring data access (http://journals.plos.org/plosone/s/data-availability#loc-acceptable-data-sharing-methods).  Data requests to a non-author institutional point of contact, such as a data access or ethics committee, helps guarantee long term stability and availability of data. Providing interested researchers with a durable point of contact ensures data will be accessible even if an author changes email addresses, institutions, or becomes unavailable to answer requests.  Before we proceed with your manuscript, please also provide non-author contact information (phone/email/hyperlink) for a data access committee, ethics committee, or other institutional body to which data requests may be sent. If no institutional body is available to respond to requests for your minimal data, please consider if there any institutional representatives who did not collaborate in the study, and are not listed as authors on the manuscript, who would be able to hold the data and respond to external requests for data access? If so, please provide their contact information (i.e., email address). Please also provide details on how you will ensure persistent or long-term data storage and availability. | 5. Author information are shared. All the related information will be shared. |
|  | 6. Please include captions for your Supporting Information files at the end of your manuscript, and update any in-text citations to match accordingly. | 6. Done. |
| Reviewer 1 | 1. Please highlight the novelty of this study in the conclusions. Discuss the clinical relevance and applicability of the study results locally & globally. | 1. See the conclusion and discussion section with comments. |
|  | 2. Two-sided unpaired t-tests or Independent Samples t Test???  The logistic regression analysis is not well described, specifically with respect to how the multivariable analysis was performed and which variables were selected for inclusion as covariates in the models | 2. T-tests and other variables are explained in the statistical analysis section. Check comment. |
|  | 3. Describe the variables used in the study in the method section State the inclusion and exclusion criteria of the case and control groups | 3. Done. Inclusion and exclusion criteria are added in the method section. |
|  | 4. Use more odds than risk in OR interpretation | 4. Resolved the issue |
|  | 5. What is missing in the discussion is that the authors should not only compare their studies with similar studies but also discuss the meaning of their findings within the context of the Bangladesh literature, which I think there are a lot. Secondly, the authors need to discuss the policy, practice and research implications of their findings. e.g what does their finding mean regarding the prevention of breast and cervical cancer risk guidelines in Bangladesh? | 5. Added more information in relation to Bangladeshi BC and CC cancer guidelines. Tried to explain all the points as suggested. Please see the comment section of the discussion. |
| Reviewer 2 | 1. Is it necessary to write The in the starting of the title? I think, word THE is not necessary. | 1. ‘The’ is removed from the title. |
|  | 2. The primer of the gene is missing. Please add the primer. | 2. Please check the supporting table 1. |
|  | 3. Please mention the name of the thermocycler with country of origin. | 3. Mentioned in the method section. |
|  | 4. In Table, the authors have mentioned the mean values but without SD. It is mandatory to mention. Please mention SD with each mean values | 4. SD values are added to the mean values. Check Table 2. |
|  | 5. Statistical analysis is incomplete. Please write according to the analysis. | 5. Statistical analysis is explained as suggested according to the analysis. |
